# Correction: Storme, G.A. Breast Cancer: Impact of New Treatments? *Cancers* 2023, *15*, 2205

**DOI:** 10.3390/cancers15184514

**Published:** 2023-09-12

**Authors:** Guy A. Storme

**Affiliations:** Department Radiation Oncology, UZ Brussel, Laarbeeklaan 101, 1090 Brussels, Belgium; guy.storme@telenet.be

There was a cited reference error in the original article [1]. A correction has been made to Section 2, Paragraph 2, Lines 5–14: “Overweight and obese patients were diagnosed later (age at diagnosis: 51.7 ± 12.0 years) than normo-ponderal ones [9]. Breast-adipose-tissue-derived mesenchymal stromal/stem cells (bASCs) were recently identified as crucial components of the tumor microenvironment (TME). Breast-cancer-associated bASCs foster malignancy of breast cancer cells by induction of epithelial–mesenchymal transition and by activation of stemness-associated genes. Mechanistic insight showed how obesity affects the phenotype of bASCs in the TME, and highlights the molecular changes inside breast cancer cells upon interaction with cancer-educated bASCs [10]. Adipose tissue of obese individuals produces inflammatory cytokines and other mediators, creating an environment that promotes cancer invasion and metastasis [11,12]”. The authors apologize for any inconvenience caused and state that the scientific conclusions are unaffected. This correction was approved by the Academic Editor. The original article has been updated.
